# Marked methylation changes in intestinal genes during the perinatal period of preterm neonates

**DOI:** 10.1186/1471-2164-15-716

**Published:** 2014-08-26

**Authors:** Fei Gao, Juyong Zhang, Pingping Jiang, Desheng Gong, Jun-Wen Wang, Yudong Xia, Mette Viberg Østergaard, Jun Wang, Per Torp Sangild

**Affiliations:** Department of Science & Technology, BGI-Shenzhen, Shenzhen, China; Department of Nutrition, Exercise and Sports, University of Copenhagen, Frederiksberg, Denmark; Department of Biology, University of Copenhagen, Copenhagen, Denmark; King Abdulaziz University, Jeddah, Saudi Arabia; The Novo Nordisk Foundation Center for Basic Metabolic Research, University of Copenhagen, Copenhagen, Denmark

**Keywords:** DNA methylation, Preterm neonates, Necrotizing enterocolitis

## Abstract

**Background:**

The serious feeding- and microbiota-associated intestinal disease, necrotizing enterocolitis (NEC), occurs mainly in infants born prematurely (5-10% of all newborns) and most frequently after formula-feeding. We hypothesized that changes in gene methylation is involved in the prenatal maturation of the intestine and its response to the first days of formula feeding, potentially leading to NEC in preterm pigs used as models for preterm infants.

**Results:**

Reduced Representation Bisulfite Sequencing (RRBS) was used to assess if changes in intestinal DNA methylation are associated with formula-induced NEC outbreak and advancing age from 10 days before birth to 4 days after birth. Selected key genes with differentially methylated gene regions (DMRs) between groups were further validated by HiSeq-based bisulfite sequencing PCR and RT-qPCR to assess methylation and expression levels. Consistent with the maturation of many intestinal functions in the perinatal period, methylation level of most genes decreased with advancing pre- and postnatal age. The highest number of DMRs was identified between the newborn and 4 d-old preterm pigs. There were few intestinal DMR differences between unaffected pigs and pigs with initial evidence of NEC. In the 4 d-old formula-fed preterm pigs, four genes associated with intestinal metabolism (CYP2W1, GPR146, TOP1MT, CEND1) showed significant hyper-methylation in their promoter CGIs, and thus, down-regulated transcription. Methylation-driven down-regulation of such genes may predispose the immature intestine to later metabolic dysfunctions and severe NEC lesions.

**Conclusions:**

Pre- and postnatal changes in intestinal DNA methylation may contribute to high NEC sensitivity in preterm neonates. Optimizing gene methylation changes via environmental stimuli (e.g. diet, nutrition, gut microbiota), may help to make immature newborn infants more resistant to gut dysfunctions, both short and long term.

**Electronic supplementary material:**

The online version of this article (doi:10.1186/1471-2164-15-716) contains supplementary material, which is available to authorized users.

## Background

Epigenetics represent stable and heritable changes in gene expression without changing the DNA sequence, providing a mechanism whereby environmental factors can affect functions of specific cells, tissues and organs [1]. DNA methylation is the best known mechanism of epigenetic modification [2] and it is particularly dynamic during the embryonic phase of mammalian development [3]. A second phase of intense DNA methylation alterations may take place during the perinatal period, when many organs and tissues undergo dramatic changes in both physiological and environmental conditions. Genome-wide epigenetic changes may also be induced by specific nutrients [4] and such methylation changes can temporarily or permanently alter the expression of tissue-specific genes, thereby supporting the short and long term maturation of tissues and organs.

Around birth, the mammalian gastrointestinal tract (GIT) has to adapt rapidly to the change in nutrition mode (from placental to enteral nutrient intake) and living environment (from a sterile life in utero to extra-uterine exposure to bacteria and viruses) [5]. The exact time frame of this critical adaptation period varies widely among the different mammalian species [5] but in pigs intestinal structure and function change markedly from ten days before birth to 3–4 days after birth. In pigs, preterm delivery at 88–95% gestation is associated with organ immaturities and clinical complications similar to those in infants born at 70–90% gestation [5]. Consequently, preterm infants and pigs lack the final maturation of the GIT and suffer from a series of intestinal defects, leading to increased risk of intestinal complications, including necrotizing enterocolitis (NEC). NEC has a high mortality and this inflammatory condition affects up to 5–10% of the hospitalized preterm infants [6]. Nevertheless, the immature GIT is able to respond to the first enteral feeding and bacterial colonization with a large increase in tissue mass and digestive capacity [5, 7, 8]. However, the responses are highly diet-dependent and even a short period of feeding with suboptimal milk formulas may trigger the events that lead to NEC: mucosal hemorrhage, bacterial overgrowth, dysfuctional immune response, and failure of multiple organs [9]. Studies of the intestinal proteome suggest that the intestinal maladaptation in preterm neonates can be explained by a feeding-induced decrease in intestinal metabolism and stress response [8, 10], but the molecular mechanisms remain unknown.

Given the high rate of preterm birth (10-15%) worldwide [11], and the difficulties in feeding preterm infants [12], it is important to know the molecular mechanisms that guide pre- and postnatal GIT maturation in preterm neonates. Previous proteomic and transcriptomic studies demonstrated that preterm and term pigs differ markedly in the intestinal response to the first feeding [8] and both diet and bacterial colonization play a role [10, 13]. Studies in pig fetuses and germ-free preterm pigs [5, 7, 13] showed that feeding-related responses occurred, even in the absence of bacterial colonization [14, 15]. Together, these data suggest that there are genome-wide, postnatal feeding-dependent changes in the gene transcription of premature gut. Due to the essential role of epigenetics in developmental regulation of gene function, a genome-wide characterization of epigenetic modifications in the preterm intestine will help to understand the mechanisms whereby the intestine of preterm neonates adapts to its new environment.

We hypothesized that epigenetic modification is a central mechanism whereby transcriptional changes occur in the intestine during the perinatal period of preterm newborns. Using our well-established newborn piglet model of intestinal adaptation and NEC, we performed a Reduced Representation Bisulfite Sequencing (RRBS) to assess the intestinal DNA methylation in four groups of piglets: 1) Full-term newborn (0d-term), 2) preterm newborn (0d-preterm), 3) healthy preterm newborns fed with infant formula (4d-preterm), and 4) preterm newborns fed with formula and showing NEC symptoms (4d-preterm-NEC). By group comparisons, we investigated the effects of advancing prenatal (PN) age (0d-term vs. 0d-preterm), increasing neonatal (NN) age (0d-preterm vs. 4d-preterm) and NEC (4d-preterm vs. 4d-preterm-NEC) on DNA methylation of intestinal genes. This initial study showed substantial descendent change of DNA methylation in a genome-wide scale that was associated with both PN and NN effects. Four genes (*CYP2W1*, *GPR146*, *TOP1MT*, *CEND1*) related to intestinal nutrient metabolism were significantly hypermethylated in their promoter CpG islands (CGIs) and showed corresponding down-regulation of transcription levels, in healthy preterm newborns fed with infant formula for four days (4d-preterm). The observed promoter hypermethylation suggests profound epigenetic effects of the first days after birth on the GIT in preterm pigs. Further studies are required to evaluate the observed methylation changes and how they vary with different diets and GIT bacterial colonization. The results may help to identify new tissue markers of GIT maturation and maladaptation-associated complications, such as feeding-induced NEC.

## Results

### RRBS data generation and characteristics of porcine intestinal methylome

To characterize the intestinal methylome of preterm piglets associated with the adaptation around birth and development of NEC, we collected empty, 2 cm full-wall sections from the mid intestine (50% intestinal length from the stomach) from four groups of newborn piglets, all delivered by caesarean section. Two groups were newborn, unfed pigs, either full-term (0d-term) or preterm (0d-preterm, each n = 2) that were sacrificed within two hours after birth. The other two groups consisted of preterm piglets fed with infant formula for four days following the rearing and feeding protocol specified previously [16], but showing severe symptoms of NEC in regions of the intestine other than the mid intestine that was selected for the analyses (4d-preterm-NEC) or being healthy (4d-preterm, each n = 3; Additional file 1: Table S1 and S2).

Genomic DNA of mid-intestines from each individual from the same group of piglets was extracted and pooled together to construct a single RRBS library, and in total four libraries were then sequenced using Illumina HiSeq2000 sequencer. RRBS was developed and widely used to measure DNA methylation of high-CG regions at single base-pair resolution [17, 18]. In two previous studies, we thoroughly evaluated the genomic coverage as well as repeatability of this technology in human samples [19, 20]. A dataset size of 5 Gb will ensure a good genomic coverage and coverage depth [19]. Furthermore, by correlating the methylation levels of commonly covered CpG sites from two independent experiments, we gained a Pearson’s coefficients of 0.98, which suggested for good technical repeatability of RRBS [20]. As this is the first time to use RRBS in the pig genome, we first performed *in silico* simulation on the genomic fragments that were enriched by digesting the pig genomic DNA with *MspI*, selecting 40–220 bp fragments and performing a 50-bp pair-end sequencing. As a result, a maximum of 3.41 million distinct CpG dinucleotides (12.17% of all CpGs) were recovered from the sequenced fragments. Similar to other mammalian genomes [20], around half (52.30%) of the CGI CpGs in the porcine genome can be enriched by RRBS (Additional file 1: Table S3). We generated a total of 23.15 gigabases (Gb) clean sequencing reads from four libraries of pooled intestinal samples (5Gb minimal for each library), reaching a minimal coverage depth of 12.5 per strand for each (Additional file 1: Table S2). By mapping the clean reads to the reference genome, we obtained a similar distribution of sequencing reads/fragments in the four libraries, indicating a highly efficient enrichment of CpG dinuceotides and relatively unbiased RRBS library construction from our experimental procedure (Additional file 1: Table S3).

After bisulfite treatment and PCR amplification, a methylated cytosine is read as “C” while a non-methylated cytosine is read as “T” in the sequencing. We calculated the ratio of total “C” reads to total sequencing reads to initially determine global level of methylcytosines (mCs) for each library with respect to the sequence context. We found that non-CG methylation was rare (<1%), compared with CG methylation (~53%), which is consistent with previous observations in mammalian somatic genomes (Additional file 1: Table S2). Besides, RRBS technology enriches high-CpG regions in the genome. Thus, we analyzed CpG methylation in the current study. Absolute methylation level for each cytosine in the library was defined as the ratio of “C” reads to total sequencing reads for a specific cytosine. We then investigated the DNA methylation level according to the chromosome features, and observed that the average methylation level correlated negatively with chromosome length (Pearson’s *r* = −0.6, *P* = 7.8e − 05), repeat density (*r* = −0.6, *P* = 7.4e − 19), GC content (*r* = −0.5, *P* = 4.7e-19) and CpG density (*r* = −0.2, *P* = 5.6e − 20). There was no obvious correlation between methylation level and gene density (*r* =0.1, *P* =2.4e − 24) or observed versus expected number of CpG (CpGo/e) ratio (*r* = −0.0, *P* =4.6e − 19) (Additional file 2: Figure S1). The negative correlation between the methylation level of individual CpGs and GC content might be explained by the observation that CpGs in the regions of high CpG density (so-called CpG islands, CGIs) [21] tended to be unmethylated, whereas CpGs in low-density regions tended to be methylated, which is consistent with previous reports in mammalian genomes [2]. These results indicated that porcine intestinal methylomes can be detected by RRBS technology with good representation, thereby ensuring accurate examination of DNA methylation alterations.

### Differential methylation patterns among four groups of piglets

**Figure 1:**
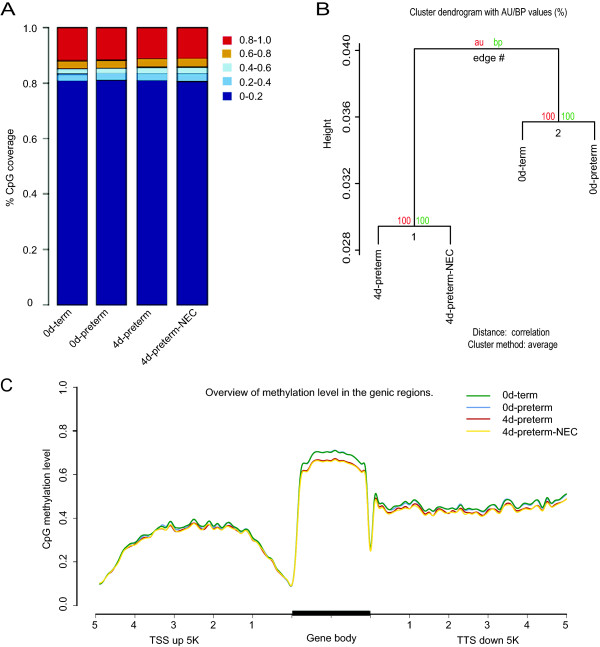
**Global DNA methylation profiles. (A)** Global pattern of CpG methylation in the four groups. The methylation levels of all CpGs were calculated and categorized into five color-coded methylation states. CpG coverage (y-axis) shows the proportion of CpGs covered with different methylation levels. **(B)** The ”pvclust” tree diagram clustering based on methylation of CpGs in the whole genome. **(C)** Average CpG methylation levels of different genetic regions. Five kilo base regions upstream and downstream of each gene were divided into 100-base pair (bp) intervals. Each coding sequence was divided into 20 intervals (5% per interval).

To examine the global pattern of the methylome, relative proportion of CpG sites, whose DNA methylation levels were classified into quintiles, were firstly depicted for each group of samples (Figure 1A). A different pattern of 5-mC distribution was observed between the preterm groups (4d-preterm, 4 d-preterm-NEC) and the newborn groups (0d-preterm, 0d-term). Especially, the proportion of highly methylated cytosines (80-100%) decreased in the preterm groups. Hierarchical clustering analysis by “pvclust” algorithm [22] on methylation levels of all genomic CpG sites also revealed a clear separation between the 4 d neonatal and 0 d newborn piglets (Figure 1B). Furthermore, the height of the “pvclust” tree diagram showed the intra-divergence within a cluster. Therefore, a higher height of the cluster with unfed, newborn groups (0d preterm and term) suggested more clear differences between these two groups than the postnatal groups (4 d preterm groups). Correspondingly, a proportional increase of low-methylated cytosines (0-20%) were observed between the 0d-term and 0d-preterm piglets, while the patterns of methylated cytosines between 4d-preterm and 4d-preterm-NEC pigs were more similar (Figure 1A). In addition, we observed that the unfed, newborn pig groups (0d) had markedly higher global methylation level than the two neonatal groups (4d) in gene bodies and downstreams. The methylation difference was 3.4% and 1.8% in gene body and downstream 5 k, respectively, while the difference in upstream 5 k was only 0.8% (Figure 1C). Considering the uneven distribution of methylation differences in different gene regions, and the similarly high bisulfite conversion rate among these libraries, as reflected by non-CpG methylation levels (<1%), this result is not likely to be due to experimental bias.

**Figure 2:**
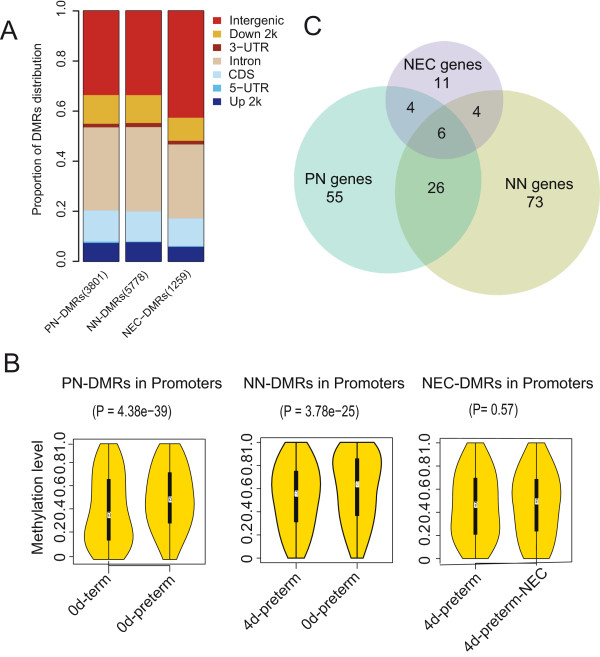
**Summary of DMRs associated with prenatal maturation (PN-DMRs, 0d-term vs. 0d-preterm), neonatal response (NN-DMRs, 4d-preterm vs. 0d-preterm) and NEC development (NEC-DMRs, 4d-preterm-NEC vs. 4d-preterm). (A)** The distribution of all DMRs identified with respect to prenatal maturation (PN-DMRs, 3801), neonatal response (NN-DMRs, 5778) and NEC (NEC-DMRs, 1259) in genetic regions. **(B)** Violin box plots show the average methylation levels of DMRs with respect to PN-DMRs, NN-DMRs and NEC-DMRs. The white points depict median values, the black boxes connect the 25th and 75th percentiles and the thin black lines connect the lower adjacent value to the upper adjacent value. The yellow area depicts a density trace, plotted symmetrically above and below the horizontal box plot. Student’s t-test was used to identify significant differences of methylation levels of DMRs located in promoters between each pairs. **(C)** Venn diagrams show the result of the cross-matching genes with DMRs overlapping with CpG island promoters with respect to PN- (blue), NN- (green) or NEC- (purple) affected genes.

Next, we carried out pair-wise comparison to identify the differentially methylated regions (DMRs) through the whole genome using a window sliding strategy [23] (see Methods). X-chromosomes were excluded from the pairwise comparison considering the DNA hypermethylation that occur during X-chromosome inactivation [24]. There was a clear PN effect (0d-preterm vs. 0d-term) on DNA methylation levels, and both these groups differed from healthy preterm piglets fed formula for 4 days (4d-preterm). The DMRs between 4d-preterm and 4d-preterm-NEC pigs differed in their susceptibility to NEC lesions. As a result, the number of DMRs was 3801 (averagely 117 bp in length, 14 CpGs) for 0d-preterm vs. 0d-term (PN effect), 5778 (averagely 122 bp in length, 13 CpGs) for 0d-preterm versus 4d-preterm (NN effect), and 1259 (averagely 79 bp in length, 11 CpGs) for 4d-preterm vs. 4d-preterm-NEC (NEC effect, Additional file 1: Table S4). More than 66% of the DMRs were distributed in intron and intergenic regions, while 7.3-8.5% of the DMRs were located within upstream 2 k to TSS regions, indicating broad but similar distribution patterns across the whole genome (Figure 2A). We then cross-matched PN- and NN-DMRs to identify the DMRs that overlapped with at least half of the CpG sites in each DMR. Interestingly, a Pearson correlation coefficient of 0.53 was obtained, showing a good correlation for most of the common CpG sites between PN- and NN-DMRs (Additional file 2: Figure S2).

### Decreased DNA methylation with different prenatal and neonatal age for genes affecting gut maturation

The GIT normally undergoes a tremendous maturation in the perinatal period in order to cope with changes in the environment [5]. In order to investigate the intestinal DNA methylation alterations prior to normal birth, we compared the groups of unfed, newborn preterm and term pigs where the majority (76.9%) of the DMRs showed decreased methylation level in term piglets, compared with preterm piglets (Additional file 1: Table S4). Further changes occurred in the first few days after birth, as shown by the comparison of 4d vs. 0d preterm pigs, where the majority of DMRs (79.6%) showed lowered methylation level in the 4d group. Among the CpG sites shared between the PN- and NN-DMRs, the majority of the shared CpG sites showed concordance of decreasing methylation due to increasing gestational age at birth and the effect of the first few days after birth (Additional file 2: Figure S2). Thus, both prenatal age at birth and the first few days after birth have strong impacts on the methylome.

As promoter hypermethylation is typically associated with the repression of gene transcription, we then focused on the genes that contained DMRs in their promoters. In total, 217 and 284 DMRs were located in the promoters of 203 and 261 genes for the PN and NN comparisons (Additional file 1: Table S4). The majority of the DMRs in promoters (74.7% and 71.8%, respectively) showed decreased methylation levels in response to advancing prenatal and neonatal age (PN and NN effects, Figure 2B). A high proportion of PN-DMRs (101 out of 203) overlapped with CGIs, corresponding to 91 genes, which were then subjected to KEGG (Kyoto Encyclopedia of Genes and Genomes) pathway and disease enrichment analysis using WebGeStalt [25, 26]. Five KEGG pathways with an adjusted P value less than 0.05 were considered as significantly enriched. These included the following pathways: glycosaminoglycan biosynthesis, oxidative phosphorylation, amyotrophic lateral sclerosis, complement and coagulation cascades and neuroactive ligand-receptor interaction. Disease enrichment analysis indicated that malfunction of these genes may lead to a broad spectrum of putative disease terms, such as pregnancy complications, hyperlipoproteinemia and type 2 diabetes (Additional file 1: Table S5). These results suggest that genes involved in intestinal metabolism, innate immunity and environmental information processing undergo very marked DNA methylation alterations during the perinatal period, reflecting an important role of DNA methylation in regulation of GIT development [27].

For NN-DMRs (0d-preterm vs. 4d-preterm), 123 out of 261 overlapped with CGIs, corresponding to 109 genes (Additional file 1: Table S4). To reveal the genes with DNA methylation alterations that were likely induced specifically by the environmental changes (including enteral feeding and gut colonization) during the first four days of neonatal life, rather than simple age effects alone, we filtered out 32 genes (Figure 2C) that were commonly identified by cross-matching PN-DMR genes with NN-DMR genes and subjected the rest, 77 genes, to KEGG pathway and disease enrichment analysis. The KEGG pathway, focal adhesion, and adhesion and subarachnoid hemorrhage diseases, were enriched from these genes (Additional file 1: Table S5).

In contrast to the DMRs associated with prenatal (PN) and neonatal (NN) age (see above), only around half (561) of the 1259 identified intestinal DMRs for NEC showed decreased methylation level in the 4d-preterm-NEC vs. the 4d-preterm piglets (Additional file 1: Table S4). In total, 49 NEC-DMRs were found located in promoters of 47 genes, 53% of which showed decreased methylation levels in the NEC piglets. Thus, about equal proportion of intestinal methylation changes, increasing or decreasing, were observed in response to NEC (Figure 2B and 2C). The 25 genes containing 27 NEC-DMRs that overlapped with CGIs. Significantly, four KEGG pathways were enriched out of these 25 genes (P value < 0.05), including calcium signaling pathway, neuroactive ligand-receptor interaction, oxidative phosphorylation and alzheimer's disease. Disease analysis showed an enrichment associated with infection and immune disease, like asthma (Additional file 1: Table S5). Interestingly, ten out of the 25 genes overlapped either with PN-DMR or with NN-DMR genes (Figure 2C), suggesting methylation of these genes was highly related to NEC outbreak, advancing prenatal age and advancing neonatal age.

### Development of BSP combined with high-throughput sequencing for methylation validation

As we only used 2–3 piglets in each group for initial genome-wide screening of methylation alterations, further validation of DNA methylation alteration of identified key genes were performed on larger sample sets. To examine the methylation of one single gene, bisulfite-sequencing PCR (BSP) combined with molecular cloning of the amplified fragments and conventional Sanger sequencing is the most routinely used method. However, the cost of deep sequencing of a large set of genes is high. Furthermore, greater variability in the methylation of CpG sites has been observed than in the direct sequencing strategy, like pyrosequencing [28]. Therefore, we developed a new BSP-based procedure by using high-throughput Illumina HiSeq sequencing (HiSeq-BSP) instead of Sanger cloning sequencing (Sanger-BSP). To do so, PCR products from multiple genes of one sample were pooled together for library construction by Illumina Pair-End protocol, and different libraries were barcoded for high-throughput sequencing using HiSeq2000 (see Methods). A deep sequencing of the PCR products can thereby be achieved for each PCR fragment cost-effectively.

**Figure 3:**
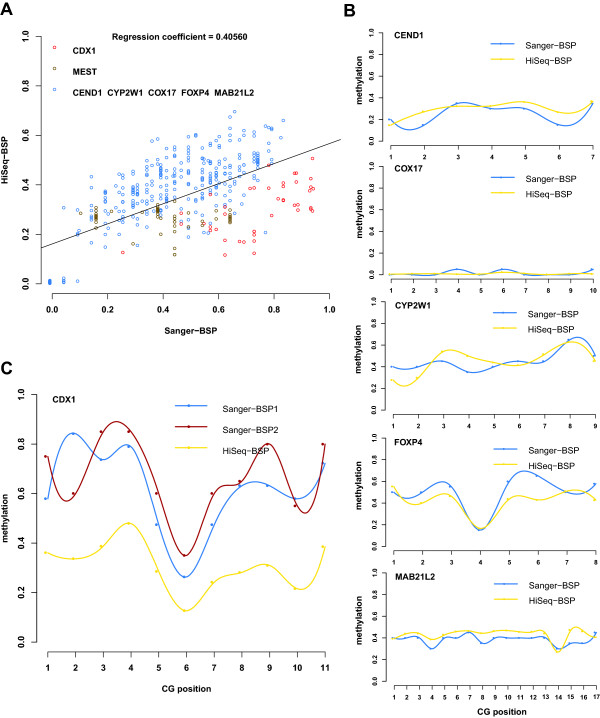
**BSP combined with high-throughput HiSeq sequencing for methylation validation. (A)** Scatter plots of the average DNA methylation level of all CpG sites covered by both HiSeq-BSP (X-axis) and Sanger-BSP (Y-axis), with each plot indicating a CpG site. A regression line is added and the regression coefficient is indicated. **(B)** Examples of DMR in the promoter of five genes (CEND1, COX17, CYP2W1, FOXP4 and MAB21L2) with general consistency for methylation levels detected by HiSeq-BSP and Sanger-BSP. **(C)** An example of DMR in the promoters of CDX1 with greater variability of methylation levels detected by HiSeq-BSP and Sanger-BSP

To evaluate the performance of this new method, we first applied both HiSeq-BSP and Sanger-BSP strategies on seven genes. Based on the uniquely mapped HiSeq sequencing reads, 80% of CpG sites on the PCR sequences could be covered with an average of 1000 fold sequencing depth (Additional file 1: Table S6), while an average of 20 clones were generated for each of the PCR fragments in Sanger-BSP. A scatter plot depicting methylation levels of all CpG sites showed a general consistency of methylation levels of CpG sites in 5 out of 7 genes between these two approaches (Figure 3A and 3B), indicating a high feasibility of the new HiSeq-BSP approach. However, Sanger-BSP tended to produce higher methylation levels than HiSeq-BSP, especially for genes, *CDX1* and *MEST* (Figure 3A and 3C). We further performed a Sanger-BSP on CDX1 in the same samples independently. We found that a second cloning procedure generated, not only a higher, but also a different methylation profile for the same gene (Figure 3C). Twenty clones might not be deep enough to secure a random selection of the PCR fragments, and the additional cloning of bisulfite-treated DNA into bacterial expression vectors might increase its variability of methylation detection. These results indicate that HiSeq-BSP had better accuracy than traditional Sanger-BSP, due to high-depth direct sequencing.

### Expression of key genes repressed by hypermethylation of CGI

We then collected mid-intestinal samples from another 24 piglets (each group n = 6) for further validation (Additional file 1: Table S1). In order to address the issue of potential inter-individual variations within the same group, we chose to examine the methylation level of key genes in each piglet, instead of a pooling strategy used in high-throughput RRBS analysis. We selected DMRs for further validations based on two criteria: First, with high co-methylation variations (difference value of methylation levels >20%), second with at least 5 CpGs and length > 30 bp. As a result, 19 PN-DMRs (18 genes), 15 NN-DMRs (15 genes) and 8 NEC-DMRs (6 genes) were successfully amplified from each of the 24 piglets and subjected to HiSeq-BSP sequencing. Considering that inter-individual variations might lead to a shift of DMR regions, we re-performed pair-wise comparison to search for DMRs on the amplified PCR fragments between two groups, 6 piglets for each group (Additional file 1: Table S6). Four (22%) PN-DMR genes, 9 (60%) NN-DMR genes and 2 (33%) NEC-DMR genes were consistently discovered with DMRs in the validation results, which all agreed with RRBS results (Additional file 1: Table S6). We calculated coefficients of variation (CV) for each CpG site among six piglets from each group, in order to determine the inter-individual variations. The CpG sites with high CVs were located within ten genes, nine of which failed the validation (Additional file 1: Table S7). The highest percentage of validation was achieved in NN-DMR genes, which might suggest that the most stable pattern of methylation changes is seen in response to the first 4 days of neonatal life in preterm neonates.

**Figure 4:**
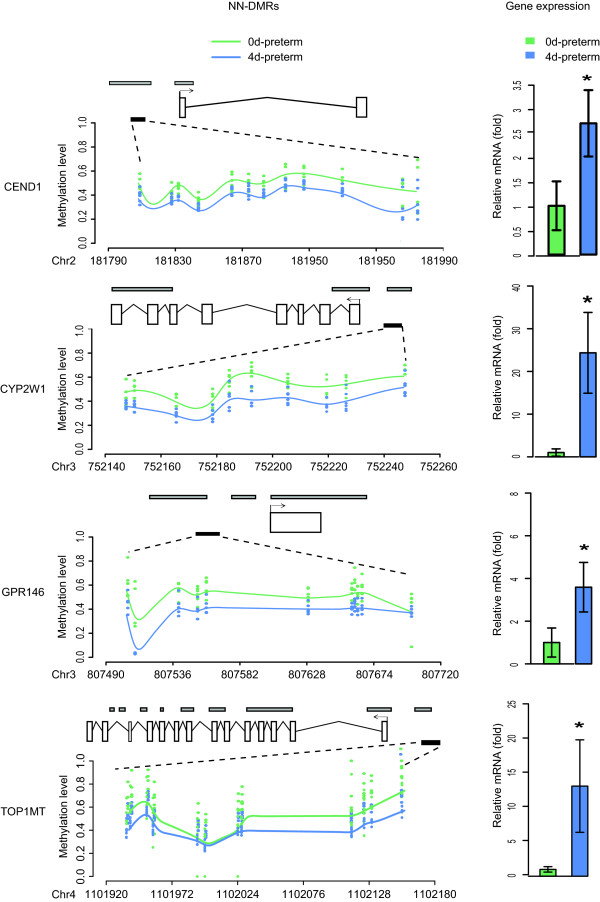
**Neonatal promoter hypermethylation associated with gene transcription repression.** After HiSeq-BSP, genes with consistent DMRs with RRBS results were subjected to real-time qPCR analysis to determine the differences of their expression levels with respect to prenatal age (PN), neonatal response (NN) and NEC disease (NEC). Four NN-genes (CYP2W1, GPR146, TOP1MT, CEND1), related to intestinal metabolism, were significantly hypermethylated in their promoter CGIs (left), and transcriptionally downregulated in the 4d-preterm compared with 0d-preterm (right). The quantitative ratios are normalized to the expression of GAPDH. The data are representative of three similar experiments and are displayed as the mean ± SD. *, *P* < 0.01 as evaluated using the Student’s t-test and with a fold change > =2.

We then tested whether the methylation changes affected the transcription level of the subset of genes with validated DMRs. Eleven genes were successfully subjected to gene expression test using quantitative RT-PCR, including three PN-DMR genes (*DEGS2*, *ADAM30* and *KLHL35*), six NN-DMR genes (*DLEU7*, *CEND1*, *RAB11FIP3*, *CYP2W1*, *GPR146*, *TOP1MT*) and two NEC-DMR genes (*CDH22* and *ZPBP*). These genes are implicated in a variety of biological processes related to organ development and intestinal functions. As a result, four NN-DMR genes (*CYP2W1*, *GPR146*, *TOP1MT* and *CEND1*) were significantly down-regulated at the transcription level (0d-preterm vs. 4d-preterm, fold change > 2, *P* value < 0.01, Figure 4), which were negatively correlated with their hypermethylation in promoter CGIs. *CYP2W1* [29], *GPR146* [30] and *TOP1MT* [31] are important for nutrient metabolism in the intestine. *CEND1* plays a role in neuronal differentiation [32], thus in development of the enteric nervous system. The down-regulation of these genes due to promoter hypermethylation may significantly affect intestinal metabolism and development, which may help to explain the difficulties of preterm neonates to tolerate enteral feeding and the down-regulation of proteins involved in intestinal metabolism and motility [8]. Among the three tested PN-DMR genes and two NEC-DMR genes, no significant difference was observed in their expression levels between the groups (Figure 4), suggesting that the effect of increasing gestational age (PN effect) and development of NEC on the expression of these genes was limited.

## Discussion

GIT maturation involves a continuous cascade of growth, differentiation, and renewal of epithelial cells [33]. Rapid GIT adaptive changes occur at the phenotypic and genome-wide transcription levels during the first few days of life in both preterm and term neonates [5]. It is only in preterm neonates, however, that the initial exposure to neonatal life may lead to intestinal dysfunction and NEC [5]. Previous studies in both preterm pigs and infants indicate that the intestinal phenotype sensitive to develop NEC is characterized by maladaptation of the intestine in response to postnatal conditions, as shown by impaired intestinal metabolism, perfusion, motility and bacterial defenses [5]. Environmental factors, coupled with impaired host-response mechanisms, appear to determine NEC sensitivity, and neither epidemiological studies in infants, nor the numerous studies in preterm pigs, suggest a large component determined by maternal or paternal genetics. Interestingly, it is mainly when infant formula, not mother´s milk, is used as the first enteral diet that the intestine fails to adapt. It is not known how the perinatal adaptation of the immature intestine involves changes at the gene level, and to which extent these changes occur prenatally or postnatally along with the introduction of milk feeding and bacterial colonization. We now show that the immature intestine undergoes rapid changes in gene methylation during the last 10 d of gestation, but even more dramatic changes occur when the immature intestine is exposed to just 4 d of formula-feeding during postnatal life. Neonatal hypermethylation of genes related to intestinal metabolism and enteric nervous system may partly contribute to the inappropriate response of immature GIT to feeding and bacterial colonization. The hypermethylation may potentially cause long-term problems of intestinal functions in preterm neonates although the effects could also be more temporary, as DNA methylation is highly dynamic throughout the lifespan of mammals [3, 34, 35]. Further studies on the long term effects of different neonatal feeding patterns may provide an answer to this important question.

We studied for the first time DNA methylation changes in a mammalian immature intestine around the time of birth. Whole tissue analyses without separating different tissue layers or cell types were performed in this study because developmental programs are often a combined result of interactions among many tissue layers and cell types. Thus, studying only one single cell type would not provide information about the changes at the whole tissue level, although the variability in results would have been less if just one cell type had been studied. Similar studies in preterm infants are impossible due to the inaccessibility of tissue samples and piglets are probably the best model to study early GIT development and disease [36] and thereby identify preventive strategies that help to avoid intestinal disease in preterm infants [37]. The results of our study highlight the importance of intestinal gene methylation during the last 10 days of gestation and the first 4 days after preterm birth, and identified several key intestinal genes that were affected. During both of these critical adaptation periods, intestinal DNA methylation increased in a genome-wide scale, indicating the repression of corresponding genes, and that this age-related methylation program was at least partly independent of the environmental changes at birth. On the other hand, the marked effects of the first 4 days of life (NN-DMRs relative to PN-DMRs) suggest strong environmental effects in addition to the predefined ontogenetic program. From our results, we cannot know whether the key neonatal adaptation factor is formula feeding, bacterial colonization or some other postnatal factors, but some previous results suggest that enteral feeding may be central for the changes. Enteral feeding alone, without bacterial colonization, can induce enormous metabolic changes in the immature GIT as germ-free fetal or preterm newborn pigs also show marked maturation after receiving enteral feeding for just 24 h [7, 38]. Intestinal dysfunction, leading to NEC, depends on bacterial colonization, but in this study we observed limited gene methylation changes in response to development of NEC (NEC-DMRs). For analyses in severely NEC-affected pigs, we deliberately selected individuals with a mid intestinal section that appeared healthy at tissue collection. This was to avoid that conclusions regarding methylation would not merely reflect the process of tissue pathology and necrosis. The results show that high sensitivity to NEC did not in itself induce profound changes in tissue methylation and we suggest that formula feeding itself is the most determining factor for intestinal gene methylation changes just after preterm birth. More studies on different feeding modes, diets and degrees of gut colonization are required to know whether intestinal gene methylation is affected by type of diet, just like NEC sensitivity. An optimal neonatal diet may promote long term health of preterm neonates by inducing permanent changes to the intestinal methylome.

The advancing gestational age at birth resulted in PN-DMRs in promoters associated with genes enriched in KEGG pathways that are considered important for gut development. “Oxidative phosphorylation” is a metabolic pathway that is indicative of mitochondrial activity and central for intestinal metabolism [39, 40], while “glycosaminoglycan biosynthesis” is key for the intestinal structure and first line defense against pathogens, and they are down-regulated in intestines affected by NEC [41, 42]. It appears that glycosaminoglycan biosynthesis is regulated by methylation changes, and after birth, mucin synthesis was also down-regulated in formula-fed pigs compared with colostrum-fed pigs [43]. The “Complement and coagulation cascades” pathway is a part of innate immunity as a nonspecific defense against pathogens [44], suggesting epigenetic changes regulates this protective mechanism during the prenatal period.

We observed a relatively low rate of DMR validation (4/15). The clear inverse relationship between DMR level and gene transcription (0/3) for a larger group size of samples from newborn preterm and term animals (PN effect) suggests high individual variation among piglets. High individual variation could also explain the relatively small average value of methylation differences in the key KEGG pathway genes between these two groups. Regardless, it is possible that the immaturity-associated DNA methylation pattern may lead to unsuccessful activation or silencing of key genes involved in normal prenatal gut development, thus helping to explain the higher risk for NEC in preterm neonates [5, 45]. For example, we observed promoter hypermethylation, and lowered expression, of the *DEGS2* gene in the newborn, preterm piglets. This gene plays an essential role in the sphingolipid synthesis in intestine [46], which in the neonatal period may be supported by milk sphingolipids [47].

In response to the first few days of neonatal life, the majority of NN-DMRs showed hypomethylation (4d-preterm vs. 0d-preterm), which is consistent with a general increase in the gene functions to support intestinal growth and functions after birth. Four hyper-methylated NN-DMR genes were identified in their promoter CGIs of the 4d-preterm pigs, and the transcription of these genes was correspondingly down-regulated. Three of these genes are related to intestinal metabolism. GPR146 is a G protein-coupled receptor, which acts as nutrient chemosensor in the intestine [48] and transmits proinsulin C-peptide signaling [49]. CYP2W1, an enzyme of the cytochrome P450 superfamily, catalyzes the reactions involved in drug metabolism and synthesis of cholesterol, steroids and other lipids [29]. TOP1MT, a mitochondrial DNA topoisomerase, is critical for mitochondrial integrity and cellular energy metabolism [31]. Decreased formula-induced expression of the above metabolic genes may disturb normal intestinal nutrient metabolism and may thereby predispose to complications. In accordance with these observations, we previously found that a switch from parenteral to enteral feeding with formula (but not mother´s milk) rapidly induces diet-dependent histopathological, functional, and proinflammatory changes in the immature intestine [9], as well as decreased expression of specific proteins involved in intestinal metabolism [8]. We now provide evidence that these immediate neonatal responses may be mediated by more fundamental changes to the genes involved, and that this may have long term consequences for intestinal functions in preterm neonates. Further studies on older preterm and term piglets, fed with different diets in the immediate postnatal period, can provide answers to these hypotheses. Potentially, the feeding time, dose, diet (infant formula or mother´s milk) and microbial colonization could exert differential effects on intestinal gene methylation. Dietary methyl donors may also be important, as shown by the observed effect of folate on DNA methylation [4]. Infant formula is very different from mother´s milk in the contents of bioactive nutrients and immunological, antimicrobial and growth-stimulating factors [50].

Development of NEC in preterm infants is characterized by a severe inflammatory response, including epithelial degeneration, edema and mucosal destruction. Our current study demonstrated that the DNA methylation changes associated with NEC were enriched in the “focal adhesion” KEGG pathway and relevant to “adhesion” and “subarachnoid hemorrhage”, suggesting that immature DNA methylation might be related to NEC. However, the pairwise comparison between healthy and NEC preterm pigs only revealed two genes with notable changes in DNA methylation levels and we could not document corresponding changes at the corresponding gene expression levels. CDH22 is a cadherin protein associated with colorectal cancer disease [51], while association of ZPBP with inflammatory bowel disease was shown in a previous genome-wide association study [52]. Despite the marked phenotypic changes in the intestine related to occurrence of NEC, no marked changes in intestinal DNA methylation was found, at least not within the first days of neonatal life.

## Conclusions

In conclusion, our data revealed marked developmental changes in DNA methylation in the immature prenatal intestine and especially during the first days after preterm birth. The marked structural and functional changes that occur in the intestine at this critical time were accompanied by large changes in the intestinal methylome, which likely induce long-lasting effects beyond the neonatal period. We speculate that enteral feeding alone can bring profound changes to DNA methylation levels for intestinal genes. Diet- and feeding-related alterations in the DNA methylation of specific genes may contribute to the high sensitivity to NEC. Further studies on intestinal DNA methylation changes, both short and long term, in response to neonatal feeding and bacterial colonization are needed. These could provide important information about the mechanisms of intestinal adaptation in all newborns, and potential targets for interventions against gut diseases in preterm newborns. Preterm birth is associated with long term complications in many organs and it is important to determine whether perinatal methylation changes can be manipulated by optimizing key environmental factors, such as diet and bacterial colonization at this critical time.

## Methods

### Animal treatment and tissue collection

Six term pigs were delivered close to full term (115–116 days gestation, by cesarean section) from two pregnant sows (Large White × Duroc, Askelygaard, Denmark), serving as the 0d-term group. Within 6 hours after birth, these pigs were euthanized (sodium pentobarbitone, 200 mg/kg, intravenous), and an approximately 2-cm-long intestinal segment left to the middle point of whole small intestine was taken from each pig, snap frozen in liquid nitrogen and stored at −80°C. This method was adopted in the following samplings of intestine. Under anesthesia, 18 preterm pigs were obtained from five pregnant sows (Large White × Duroc; Askelygaard, Denmark) by cesarean delivery at 90-92% (104–107 days) gestation, as described earlier [38]. Six of these pigs were euthanized within 6 hours after birth without any feeding (0d-preterm group). The intestine segments were collected and frozen at −80°C. The remaining 12 preterm pigs were reared individually in infant incubators (Air-Shields, Hatboro, PN) and fitted with orogastric catheters for parenteral nutrition and orogastric feeding tube. The pigs were given parenteral nutrition at a rate of 4 mL/(kg × h) on d1 and 6 mL/(kg × h) on d2 as well as minimal enteral nutrition: 24 mL/(kg × d) on d1 and 40 mL/(kg × d) on d2 with infant formula. The parenteral nutrition was based on a commercially available product (Kabiven, Fresenius Kabi) and adjusted in nutrient composition to meet the requirement of pigs. On d3, they were transferred to full enteral nutrition [120 mL/(kg × d)] for two more days before sacrifice. The mid intestine was also dissected out and frozen at −80°C. Detailed feeding protocol was previously described [16]. Pigs were continuously monitored for clinical symptoms of NEC and at tissue collection the severity of NEC-like intestinal lesions was evaluated using a scoring system previously described [16]. Six pigs remained healthy at the time of tissue collection on day 4 (4d-preterm) while six other pigs had significant NEC lesions in their intestines (4d-preterm-NEC). For validation experiments, mid intestine samples from other 6 piglets for both groups were collected, which were treated in the same way in RRBS study. Information on all the samples is included in Additional file 1: Table S1. All the procedures on animals were approved by the National Committee on Animal Experimentation in Denmark (permit no. 2012-15-2934-00193).

### The RRBS library construction

For each intestine sample, total DNA was prepared by proteinase K/phenol extraction and examined by agarose gel electrophoresis. Then, the extracted DNA of all intestine samples within each group were pooled together for RRBS library construction, thereby four pools of intestinal genomic DNA were prepared for the four groups of piglets (Additional file 1: Table S1), respectively, and used for RRBS library construction as previously described [19]. Briefly, four μg of genomic DNA was digested with 100U of *MspI* enzymes (NEB) in 100 μL reactions at 37°C overnight. After purification, the digested products were progressed by blunt-ending, dATP addition and methylated-adapter ligation. Two ranges of 160-240 bp and 240-340 bp fractions were excised from a 2% agarose gel, respectively. Then bisulfite conversion was conducted by ZYMO EZ DNA Methylation-Gold Kit™ (ZYMO) following the manufacturer’s instructions. The converted DNAs were amplified using JumpStart™ Taq DNA Polymerase (Sigma) by 11–13 cycles (11 cycles for 160–240 bp and 13 cycles for 240–340 bp) of 94°C for 30 sec, 58°C for 30 sec, 72°C for 30 sec. RRBS libraries were then analyzed by Agilent 2100 Bioanalyzer (Agilent Technologies) and quantified by PCR.

### RRBS sequencing and data processing

The RRBS libraries were sequenced using Illumina HiSeq2000 Analyzer according to the manufacturer’s instructions. Raw sequencing data was processed by the Illumina base-calling pipeline. Low-quality reads that contained more than 30% ’N’s or over 10% of the sequence with low quality value (quality value <20) per read were omitted from the data analysis. The pig reference genome (Sscrofa10.2) by Swine Genome Sequencing Consortium (SGSC) was downloaded from Genbank database (ftp://ftp.ncbi.nih.gov/genbank/genomes/Eukaryotes/vertebrates_mammals/Sus_scrofa/Sscrofa10.2/). The annotation was downloaded from Ensemble (Sus_scrofa.Sscrofa10.2.67.gtf.gz). The clean reads were aligned to the reference genome using SOAP aligner (Version 2.01) [53] according to a previously published method [23]. The uniquely aligned reads that contained *MspI* enzyme digestion site in the ends were used for further analysis.

### Identification of DMRs, KEGG pathway and disease enrichment analysis

To identify putative DMRs, a sliding window strategy was used as previously described with minor adjustments [23]: First, commonly covered CpG sites with sequencing depth > =5X between two groups were selected as candidate sites. After bisulfite treatment and PCR amplification, a cytosine will be read either as “T” if it’s unmethylated or “C” if it’s methylated, following binomial distribution, as previously suggested [54]. Methylation level of individual cytosine can then be defined as the ratio of “C” counts to total counts of “C” and “T” in the sequenced reads for each individual cytosine. Therefore, a two-tailed Fisher’s Exact Test was first used to test the “C” and “T” counts for each cytosine between two groups. Then, the first CpG with significantly differential methylation (automatically corrected P-value < 0.05) was used as an initial locus of DMR, and following candidate sites were merged into a candidate DMR according to following criteria: First, the distance between two neighboring candidate sites < = 300 bp, in order to cope with *MspI*-digestion based RRBS strategy. Second, all candidate CpG sites in the candidate DMR maintain the same methylation direction (hyper- or hypo-). Third, a candidate DMR must harbor 5 or more candidate CpG sites, among which at least 3 CpG sites should have Fisher’s exact test P-value less than 0.05. Last, for each of above candidate DMRs, a Fisher’s exact test was performed again based on the mean “C” and “T” counts for all the CpG sites within the candidate DMRs. A false discovery rate (FDR) adjustment was then performed on the P-values, using the R package of “P.adjust” that is based on BH method [55]. DMRs with Fisher test FDR P-value <0.05 and difference of mean methylation levels between two sample < 20% were taken as statistically significant. KEGG pathway and disease enrichment analysis was performed using the WebGestalt web server (http://bioinfo.vanderbilt.edu/webgestalt/) [25, 26].

### Validation by bisulfite sequencing PCR combined with cloning and Sanger sequencing

PCR primers were designed using the online MethPrimer software (http://www.urogene.org/methprimer/index.html) and listed in Additional file 1: Table S8. Genomic DNA (500 ng) was converted using ZYMO EZ DNA Methylation-Gold Kit™ (ZYMO) and one-tenth of the elution products were used as templates. PCR amplification was carried out with a thermal cycling program of 94°C for 1 min, 30 cycles of 94°C for 20 sec, 50 ~ 60°C for 30 sec, 72°C for 40 sec and then final 4 min incubation at 72°C. For Sanger sequencing, PCR products were purified using the QIAquick Gel Extraction Kit (Qiagen) and subcloned, 20 colonies from each product were sequenced using the 3730 Genetic Analyzer (Applied Biosystems).

### Validation by bisulfite sequencing PCR combined with HiSeq sequencing

PCR primers were designed using the online MethPrimer software (http://www.urogene.org/methprimer/index.html) and listed in Additional file 1: Table S8. For Hi-Seq sequencing, PCR products of multiple genes from one sample were quantified by Tangen gel system and pooled together equally. One μg of pooled PCR products was fragmented by a Covarias sonication system to a mean size of approximately 200 bp followed by end-polishing, A-tailing, adapter ligation and 5-cycle PCR to generate a barcoded library. Barcoded libraries from different samples were then pooled together equally and used for cluster generation and standard pair-end sequencing with 50 bp reads (PE50) using Illumina HiSeq 2000. After sequencing, the Illumina reads were post-processed and aligned to the pig reference regions (all PCR regions) using SOAP aligner (Version 2.01) [53] according to a previously published method [23] with default parameters that excluded reads with more than five mismatched bases. Multiple reads mapping to the same position were counted only once to remove potential bias from PCR.

### Quantitative real time PCR

Total RNA was isolated using Qiazol and RNeasy Midi kit (Qiagen), and cDNA was synthesized using 500 ng total RNA in a total of 20 μl reaction mixture using the QuantiTect Reverse Transcription kit (Qiagen) according to the manufacturer’s instructions. Primers for real-time quantitative PCR (RT-qPCR) were designed using Primer 5 software and listed in Additional file 1: Table S8. Real-time qPCR analysis was performed on ABI Prism 7700 (Applied Biosystems, Tokyo, Japan) using SYBR Green real-time PCR master mix (Toyobo Co., Japan). Relative expression levels of objective mRNAs were calculated using the ∆∆Ct method and normalized to GAPDH. All data are presented as the mean values ± S.E. Comparisons were made using the Student’s t-test and a two-sided p-value < 0.05 was considered to indicate statistical significance.

### Data availability

All RRBS sequencing and processed data was deposited in the Gene Expression Omnibus (GEO) with accession GSE53747. Validation data was deposited in GigaDB, the GigaScience database, with the unique identifier doi:10.5524/100043 [56].

## Electronic supplementary material

Additional file 1: **This file contains Table S1-S8.** (XLSX 1 MB)

Additional file 2: **This file contains Figure S1-S2.** (DOC 256 KB)
