# Supplementary material for: Marked methylation changes in intestinal genes during the perinatal period of preterm neonates
Source: BMC Genomics. 2014 Aug 26;15(1):716. doi: 10.1186/1471-2164-15-716 (PMC4153944; doi:10.1186/1471-2164-15-716)
Supplement: Supplementary file 2 — Additional file 2: This file contains Figure S1-S2. (DOC 256 KB) [file 12864_2014_6393_MOESM2_ESM.doc]

**Figure S1**


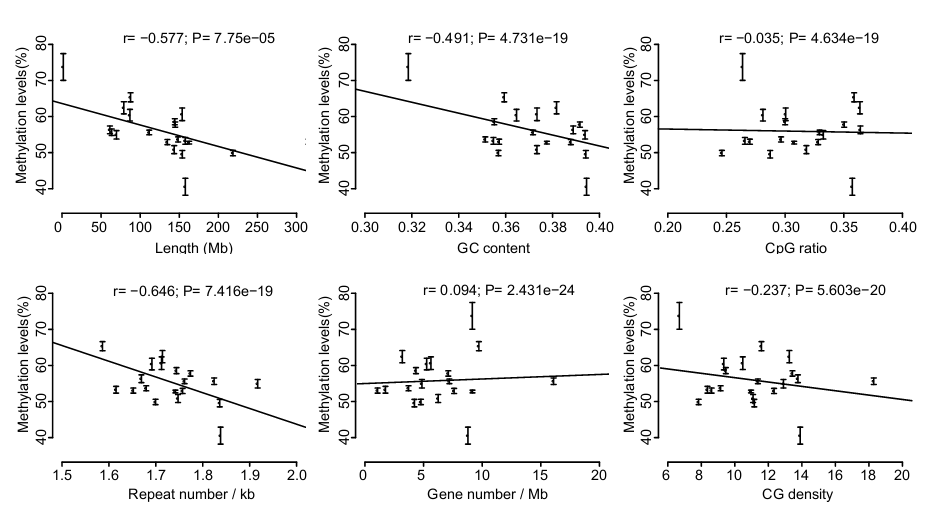


**Figure S1. DNA methylation level in relation with genomic features.** The Pearson’s correlation between CpG methylation levels and features of pig chromosomes (n = 20). The CpG methylation level (%) was plotted against the length, GC content, CpG(o/e) ratio, repeat density, gene density, and CpG density of individual chromosome, in which means ± SD (n = 4) were presented. Line represents linear regression.

**Figure S2**


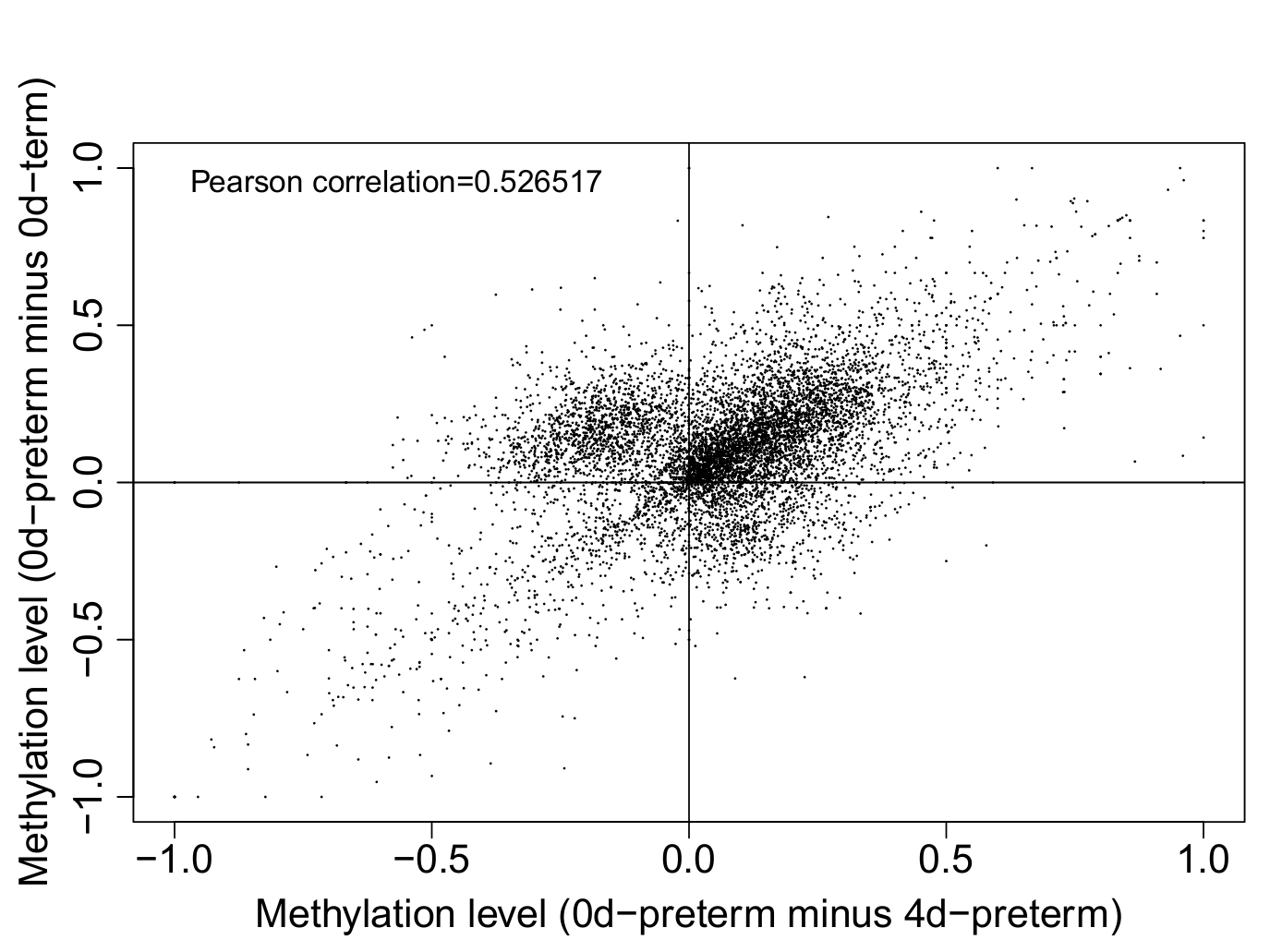


**Figure S2** Scatter plots of methylation levels in shared CpG sites between PN- and NN-DMRs. The methylation level difference between 0d-preterm and 4d-preterm or between 0d-preterm and 0d-term was indicated in X axis or Y axis, respectively. The coefficients of Pearson correlation was marked.
